# Polymerase Chain Reaction for Screening Blood Donors at Risk for Malaria: Safe and Useful?

**DOI:** 10.3201/eid0808.020025

**Published:** 2002-08

**Authors:** T. Hänscheid, E. Valadas, M.P. Grobusch

**Affiliations:** * Faculty of Medicine, Hospital de Santa Maria, Lisboa, Portugal; † Institute of Tropical Medicine, Tuebingen, Germany

**To the Editor:** Transfusion-transmitted malaria, although extremely uncommon in most countries not endemic for malaria, may have fatal consequences if undetected (1,2). Benito and Rubio (3) addressed this timely issue by presenting data on screening of blood donors at risk for malaria in Spain with a seminested polymerase chain reaction (SNM-PCR). Of 125 donors at risk (immigrants from malarious areas), these researchers identified five cases of *Plasmodium falciparum* by using SNM-PCR with a 5-mL EDTA blood sample. Benito and Rubio’s conclusion was that the SNM-PCR could serve as the reference test for screening blood (3). Obviously, this conclusion implies the potential use of blood donations from donors who were at risk but whose PCR results were negative. We believe that this practice would be dangerous and could lead to the administration of unsafe blood.

The PCR, like any method based on direct detection of the parasite, does have a shortcoming: the amount of specimen processed determines the limit of detection. Even if the described PCR method detected a single parasite in the 5-mL blood sample used by the authors (3) (hypothetical sensitivity 0.0002 parasites/µL), a standard 450-mL blood donation could still contain <90 parasites and have a negative PCR result. However, with the “best” sensitivity reported by Benito and Rubio (3) of 0.004 parasites/µL, a standard 450-mL blood donation could contain <1,800 parasites and still be tested negative by SNM-PCR. Surely, <1,800 parasites is enough to cause disease in a blood recipient. As few as 10 parasites per donation (perhaps even fewer) may cause disease. Theoretically, any method would have to detect a single parasite per unit of blood to be safe, thus requiring a hypothetical detection limit of 2.2x10^-6^ parasites/µL. However, a sample equal to the unit of donated blood (450 mL) would have to be processed to achieve this level. In addition, one would have to assume that parasites are equally distributed in the peripheral blood at the time of donation.

Little is known about the frequency of very low parasitemias. No large-scale epidemiologic studies have been conducted in which large amounts of blood (e.g., the equivalent of a blood donation or 450 mL) were collected. The authors (3) could not confirm the PCR-positive cases by microscopy. This finding suggests very low levels of parasitemia, below the sensitivity of thick smears, in the range of 1–20 parasites per μL (4). Similar results were observed in blood donors associated with transfusion-transmitted malaria in the United States, in which malaria smears were positive in only 17 of 49 donors (1). PCR is similar to microscopy in screening donors at risk, even if the detection limits are different. Hommel and Gilles report in Topley and Wilson that for disease-endemic countries “the use of PCR… …is not, despite its much increased sensitivity, a complete guarantee of safe blood, because the absence of parasites in a 20-µL sample does not exclude the possibility of infection in the remaining volume of the 450 mL blood unit” (5).

On the other hand, one might argue that screening the whole blood supply for malaria by PCR may detect the rare blood donation with undetected malaria, with higher parasitemias. However, the generally accepted deferral criteria for blood donors at risk seem highly efficient. In the United States, only 14 cases of transfusion-transmitted malaria were reported from 1990 through 1999 (1). The same authors estimate that this deferral policy led to 50,000 rejected donations in a total of 13 million per year (0.3%). At an estimated expense of $2.00–$3.00 per PCR, a general screening program would cost more than $20 million–$30 million per year. Each case of malaria prevented would therefore cost in excess of several million U.S. dollars.

Several novel diagnostic methods have been developed recently (6). However, we agree with Mungai et al. (1). These methods, including PCR, have still not been shown to detect the lowest possible parasitemia that can cause malaria. Showing that a method is able to detect donors at risk for malaria, as done by Benito and Rubio (3), is insufficient. On the contrary, the only convincing study design would be to show that donors at risk who have a negative PCR result also do not harbor parasites and cannot transmit the disease. Accordingly, careful screening of blood donors in nondisease-endemic countries, in accordance with the established exclusion criteria, remains the best way to prevent transmission of malaria (1,2).
